# Impact of Hepatitis C Treatment as Prevention for People Who Inject Drugs is sensitive to contact network structure

**DOI:** 10.1038/s41598-017-01862-6

**Published:** 2017-05-12

**Authors:** Cornelia Metzig, Julian Surey, Marie Francis, Jim Conneely, Ibrahim Abubakar, Peter J. White

**Affiliations:** 1 MRC Centre for Outbreak Analysis and Modelling and NIHR Health Protection Research Unit in Modelling Methodology, Imperial College London School of Public Health, London, W2 1PG UK; 2 Department of Mathematics, Imperial College London, London, SW7 2AZ UK; 3 Research Department of Infection and Population Health, University College London, London, WC1E 6JB UK; 4 Hepatitis C Trust, 27 Crosby Row, London, SE1 3YD UK; 5 TB Section, National Infection Service, Public Health England, London, NW9 5EQ UK; 6 MRC Clinical Trials Unit, University College London, London, WC2B 6NH UK; 7 Modelling and Economics Unit, National Infection Service, Public Health England, London, NW9 5EQ UK

## Abstract

Treatment as Prevention (TasP) using directly-acting antivirals has been advocated for Hepatitis C Virus (HCV) in people who inject drugs (PWID), but treatment is expensive and TasP’s effectiveness is uncertain. Previous modelling has assumed a homogeneously-mixed population or a static network lacking turnover in the population and injecting partnerships. We developed a transmission-dynamic model on a dynamic network of injecting partnerships using data from survey of injecting behaviour carried out in London, UK. We studied transmission on a novel exponential-clustered network, as well as on two simpler networks for comparison, an exponential unclustered and a random network, and found that TasP’s effectiveness differs markedly. With respect to an exponential-clustered network, the random network (and homogeneously-mixed population) overestimate TasP’s effectiveness, whereas the exponential-unclustered network underestimates it. For all network types TasP’s effectiveness depends on whether treated patients change risk behaviour, and on treatment coverage: higher coverage requires fewer total treatments for the same health gain. Whilst TasP can greatly reduce HCV prevalence, incidence of infection, and incidence of reinfection in PWID, assessment of TasP’s effectiveness needs to take account of the injecting-partnership network structure and post-treatment behaviour change, and further empirical study is required.

## Introduction

Hepatitis C Virus (HCV) infection among people who inject drugs (PWID) is a public health priority, with prevalence in London being around 43%.[Aldridge *et al*. submitted] With the advent of direct-acting antivirals (DAAs), which are more tolerable and have higher efficacy than previous therapy, with cure rates up to 90%^1^, Treatment as Prevention (TasP) for HCV in PWID has received attention^2–5^. However, assessing the potential benefits of TasP is challenging because it depends greatly on the patterns of transmission, which are not yet well understood.

The effectiveness of TasP for HCV has been analysed with compartmental models (e.g.refs 5 and 6) in which individuals are assumed to share injecting paraphernalia at equal rate with everyone, making the population “well-mixed”. Recently, more-realistic models have considered the injecting-partnership network of PWID, simulating HCV transmission on exponential random networks^7^ or on empirical networks determined by respondent-driven sampling^8, 9^. However, it remains difficult to infer to the structure of the full network, as respondent-driven sampling generally overestimates small loops^10^, and often questionnaires have limited the maximum number of injecting partners that could be reported, so truncating the reported frequency distribution^9, 11^. Furthermore, these networks models are all static and so do not incorporate entry into and exit from the network of PWID, or changes over time in individuals’ injecting partners, which will be important for transmission dynamics since they occur on a similar timescale to the duration of infection^12^.

Reinfection following treatment would clearly reduce the effectiveness of TasP, and therefore its frequency is an important consideration. Evidence from the US, the Netherlands, Norway and Canada^13–17^ suggest that reinfection risk after treatment of HCV infection is significantly lower than the risk of initial infection, indicating a change in the behaviour of individuals to reduce their risk. However, there is wide variation in reported reinfection rates after treatment^18^. Importantly, reinfection risk is determined by both the behaviour of treated individuals and the prevalence of HCV in their injecting partners (if they continue to inject drugs post-treatment). Therefore, if treatment were applied with high coverage then the reductions in prevalence would reduce reinfection frequency below the rates observed in small-scale trials. The magnitude of this population-level effect is expected to depend upon network structure and rates of treatment in the population, which we explore.

In this paper we introduce a model for a dynamic injecting network, which we use to study HCV transmission and the impact of TasP. Individuals enter and exit the system and change injecting partners, whilst the degree distribution and summary statistics remain stationary^19^ (see Table 1). The model structure and parameters are informed by data from a behavioural and demographic survey of PWID in London, England, a prevalence survey, [Aldridge *et al*. submitted] and other topological features of social networks^20^. We study the effectiveness of TasP at different coverage levels in our network model, compared with TasP on two simpler (but also dynamic) networks, as well as a compartmental model, to assess the impact of the network characteristics on TasP. We examine pessimistic and optimistic scenarios regarding the effect of post-treatment behaviour change preventing reinfection. We use several measures of TasP effectiveness: reduction in HCV prevalence and incidence, infections averted and life-years gained.

**Table 1:** Comparison between the skewed-clustered-assortative network and random network (*λ_*max*_ being the largest eigenvalue of the adjacency matrix, **<k> the degree).

	Exponential-clustered network	Exponential network	Random network
Number of nodes (i.e. individuals in the network)	1000	1000	1000
Mean degree (i.e. number of partners per individual) at one time-point (90% credible interval)	3.7 (3.5–3.9)	3.62 (3.4–3.86)	3.7 (3.6–3.8)
Average path-length (i.e. number of ‘steps’ between two individuals in the network)	6.1	4.5	6.9
Clustering coefficient^41^ (90% credible interval)	0.36 (0.34–0.38)	0.22 (0.2–0.24)	0.065 (0.06–0.075)
Assortativity^20^ (standard deviation)	0.016 (0.011–0.021)	0.03 (0.02–0.04)	0.0007 (0.0005–0.0009)
1/*λ*_*max*_ of static network*	0.00407	0.0045	0.00225
<k>/<k^2>**^42^	0.113	0.101	0.204
Epidemic threshold for *β* (with heterogeneous mean field)**^42^	2.0	2.0	1.0
Transmission probability per partnership and month, *β*	0.053	0.053	0.068

## Results

### Network structure and model calibration

**Figure 1:**
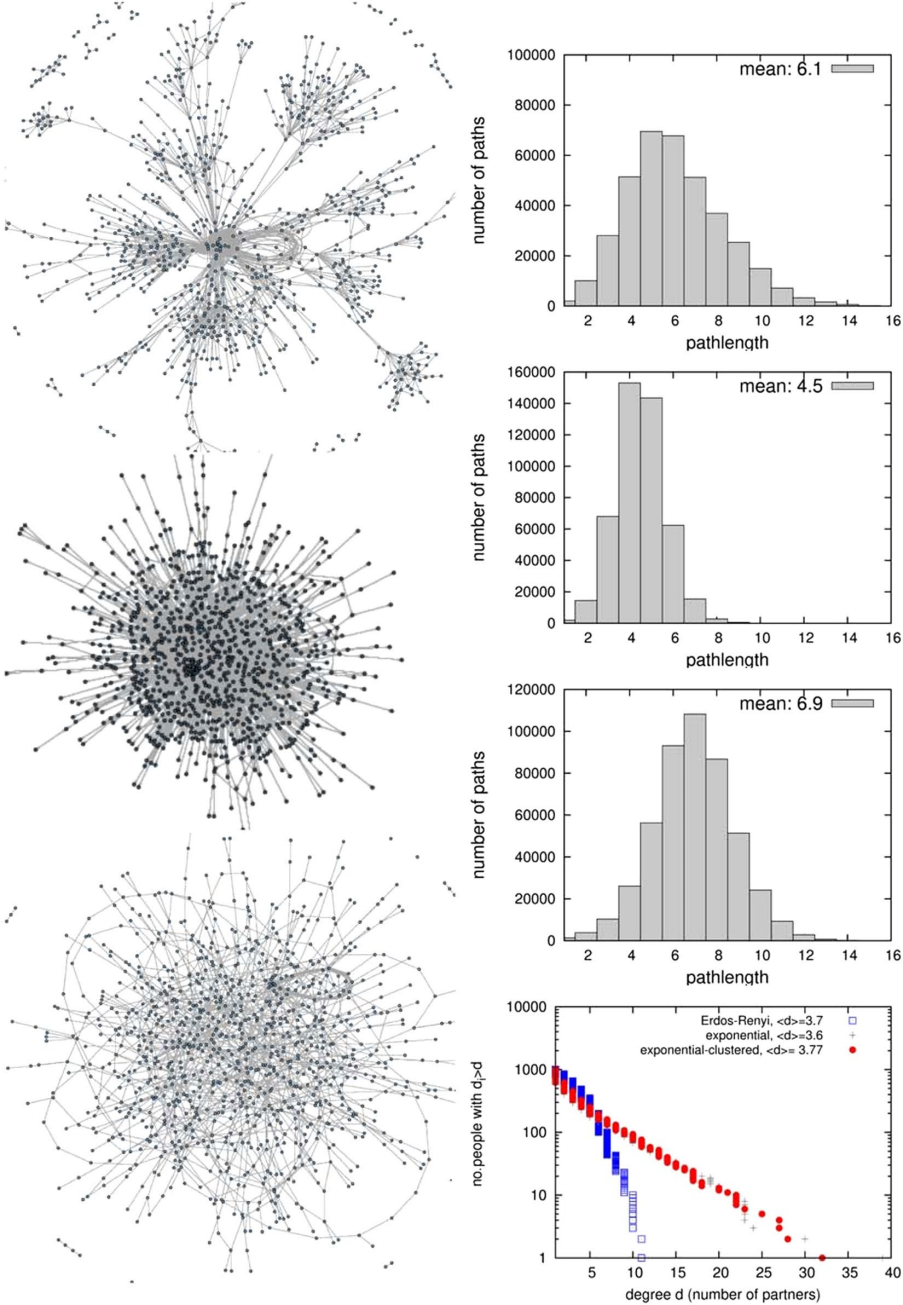
The three networks compared in this analysis. Right column: distribution of path-lengths in the network, and average path-length Inlay: Counter-cumulative representation of the degree distributions of the three networks in log-linear scale, where a straight line corresponds to an exponential distribution (both for the counter-cumulative distribution and for the probability density, which is its derivative).

Figure 1(a) shows the ‘exponential-clustered’ (ec) network we developed, which exhibits a number of features found in real social networks:^21^ an exponential degree distribution (which means that some individuals are highly connected)^20, 22^, positive clustering^9, 23^, positive assortiveness (degree correlations)^20^ and short average pathlength (i.e. number of ‘steps’) between individuals. To understand the effect of these features for HCV transmission and TasP, we compare it to TasP on two simpler networks: an exponential (unclustered) network and a (even simpler) Erdös-Renyi random network, shown in Fig. 1(b) and (c). To calibrate the prevalence in the random network to match the observed level, the transmission rate per network link was adjusted (see Table 1).

**Figure 2:**
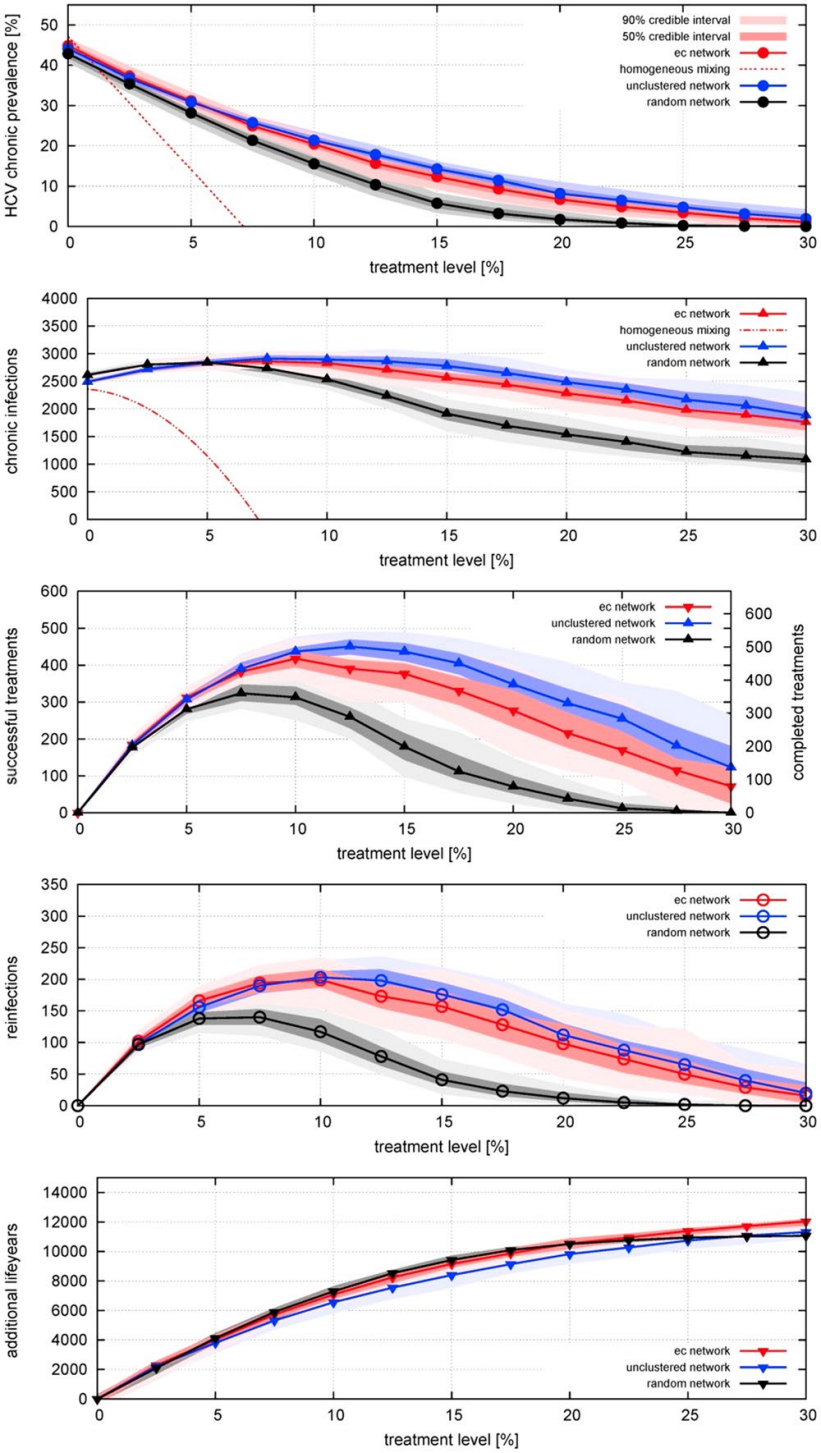
Effect of different treatment rates on equilibrium (**A**) prevalence of HCV infection; incidence per year of (**B**) chronic infection (including arising from reinfection), (**C**) cured by successful treatment, (**D**) reinfection; and (**E**) life-years gained; for three different network structures, for treatment rates varied from 0 to 30% per year, in the absence of behaviour-change post-treatment. Points indicate median results and shading represents the 50% range (i.e. inter-quartile range) and 90% ranges of 100 simulations of a network with 1000 PWID over a time period of 20 years (i.e. turnover of 50 people entering and exiting per year). For prevalence and incidence, the behaviour of the homogeneous-mixing model is also shown: as it is a deterministic model there is no range of uncertainty.

**Figure 3:**
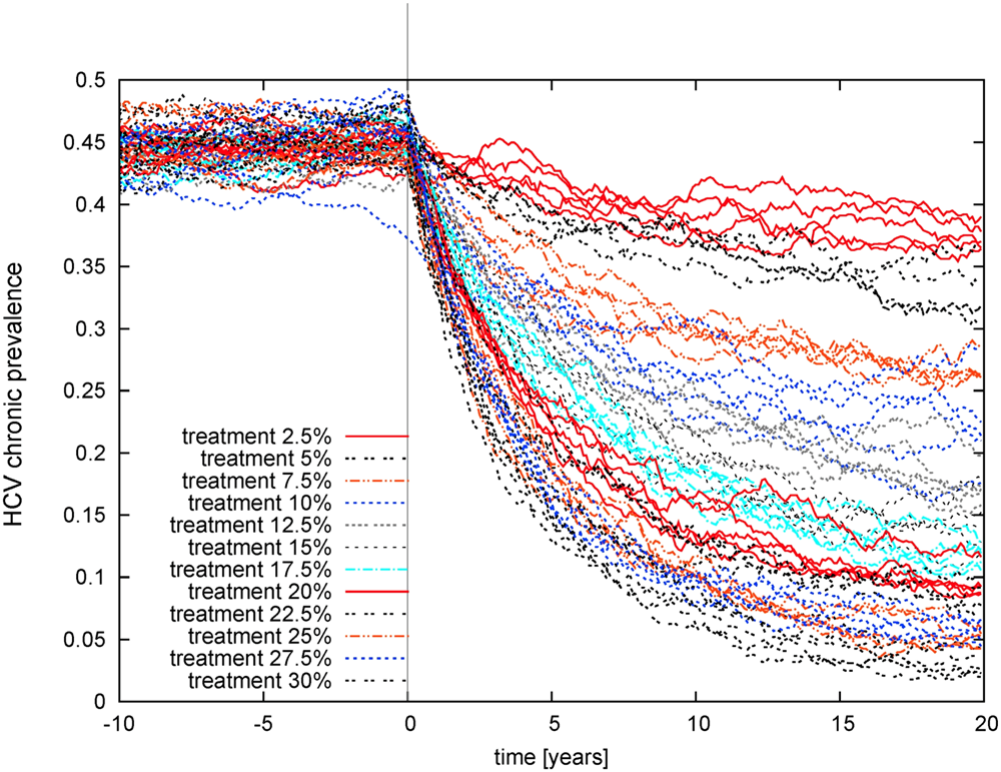
Prevalence of chronic infection over time following the introduction of treatment at different levels ranging from 2.5% to 30% on the same ec-network as in Fig. 2. The system is at equilibrium before treatment is introduced at time 0. Results of individual simulations are shown.

The short average pathlength of the ec-network facilitates HCV transmission to uninfected individuals who join the network and to infected individuals who have been treated, leading to high incidence and high frequency of reinfection (despite a lower transmission rate per contact). Conversely, clustering increases path-length (and therefore lowers prevalence) for a given degree distribution (see Fig. 2), and lowers prevalence and infections. This paper focuses on the stationary state (transient effects of TasP introduction are shown in Fig. 3, which shows that the majority of the reduction in prevalence occurs over the first ~5 years).

### Effect of treatment in different networks

We consider initially a scenario in which PWID who have been successfully treated for HCV can get re-infected (Fig. 2). In all three of the networks, prevalence declines as coverage is increased from zero. These reductions in prevalence are reflected in life-years gained by the cohort of PWID, which increases markedly as coverage increases (Fig. 2e). Incidence increases at very low levels of coverage, due to treatment increasing the number of susceptibles who can then become (re)infected, but incidence peaks and declines at higher treatment levels, due to the reduction in prevalence. On the ec-network there is relatively little decline in incidence as coverage is increased to high levels. Incidences are even higher on an exponential unclustered network, and lower on a random network.

The effectiveness of TasP is also compared to a homogeneous-mixing compartmental model of SIS-type with population turnover (see equations 1 to 7). Here, in contrast to the network models, a relatively low incidence produces a 43% prevalence level in absence of treatment, and a relatively low coverage suffices to bring prevalence down to zero. The reason for the discrepancy in prevalence is the low number of neighbours in networks, and a network-dynamic effect: the number of someone’s partners in dynamic networks, although random, increases on average with time spent in the network. People enter the system uninfected and with only one partner, but by the time they become infected and receive treatment they are more connected than the system average, and so can be reinfected more readily. In contrast, in a homogeneously mixed population, the number of infected neighbours is equal for all, and there is no notion of time to infection: everyone is connected to everyone (although the population is represented in aggregate, not as discrete individuals). This means that as soon as someone becomes infected everyone who is uninfected has an increased risk of becoming infected, hence the low incidence required to produce the observed prevalence. Conversely, as soon as someone is cured by treatment, everyone who is uninfected has a reduced risk of becoming infected, hence the large impact of TasP in reducing reinfection risk and reducing prevalence.

Heterogeneity from turnover in partnerships is more pronounced for the (slowly decaying) exponential degree distributions, and is the reason why the initial increase in infections occurs even at higher treatment rate for a more skewed network. For high coverage, prevalence is reduced sufficiently so that infections also decline (see Fig. 2b and c).

Additionally, the number of reinfections (Fig. 2d) rises and has a peak at some level of coverage before declining again. This is not a network effect but is also present in the compartmental model, (equation 7), as re-infections are proportionate both to the (rising) number of treatments and to the (declining) prevalence. In networks, in those with shorter average path-length, reinfections happen more frequently, and a high treatment level is required to reduce them.

### Effect of post-treatment behaviour change preventing reinfection

**Figure 4:**
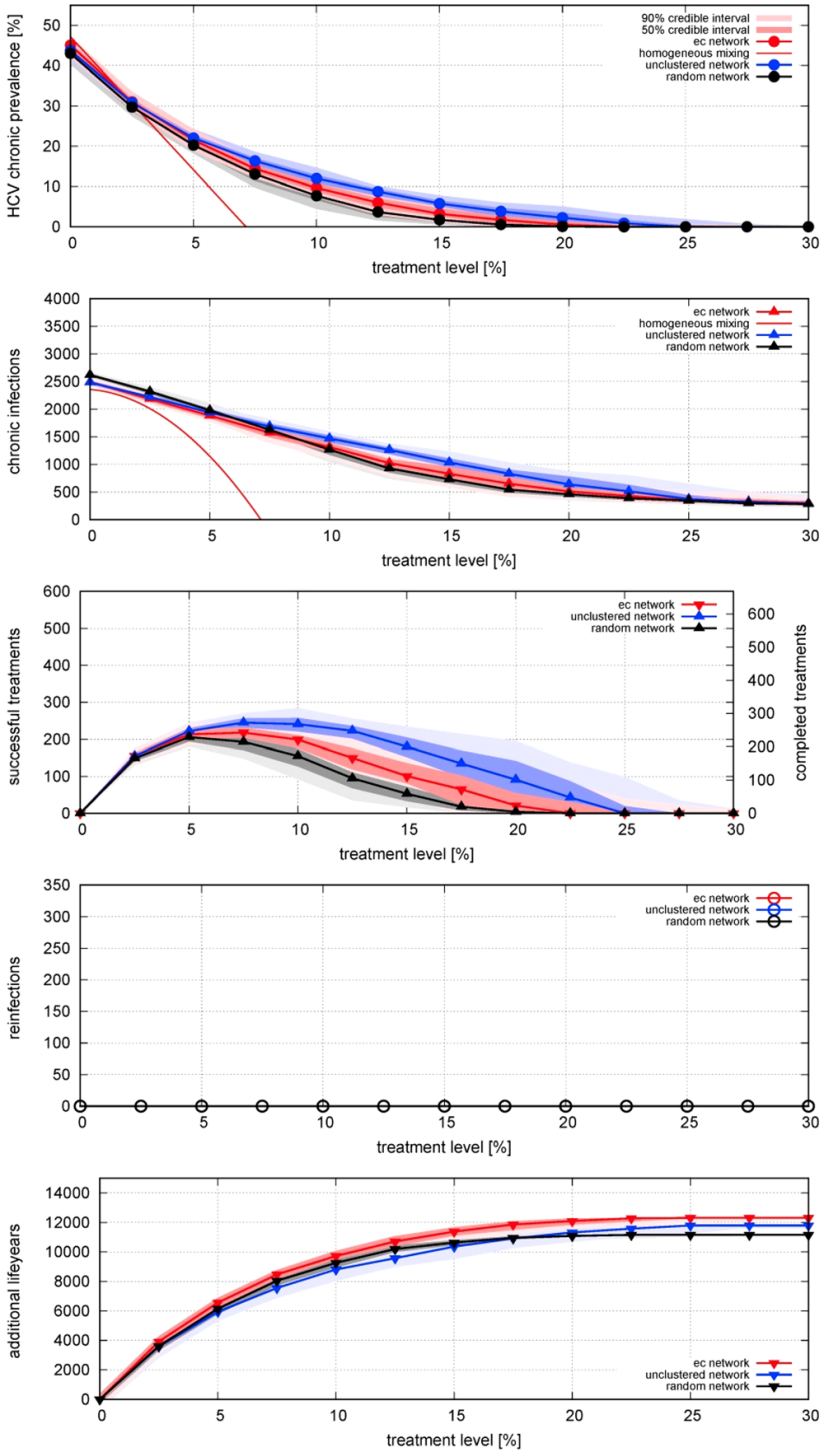
Effect of different treatment rates on equilibrium (**A**) prevalence of HCV infection; incidence per year of (**B**) chronic infection, (**C**) cured by successful treatment, (**D**) reinfection – which is always zero in this scenario because cured individuals change their behaviour to eliminate their reinfection risk; and (**E**) life-years gained; for three different network structures, for treatment rates varied from 0 to 30% per year, with behaviour-change post-treatment preventing reinfection. Points indicate median results and shading represents the 50% range (i.e. inter-quartile range) and 90% ranges of 100 model realizations of a network with 1000 PWID over a time period of 20 years (i.e. turnover of 50 people entering and exiting per year). For prevalence and incidence, the behaviour of the homogeneous-mixing model is also shown: as it is a deterministic model there is no range of uncertainty.

Now we examine an optimistic scenario in which behaviour change post-treatment eliminates the risk of reinfection (Fig. 4). Numbers of infections and therefore required numbers of treatments are much lower than in the previous scenario, since the pool of susceptibles is smaller as cured individuals do not return to it (Fig. 4). This holds for all networks.

This difference in prevalence and incidence between the scenarios is highest for the networks with short average path-lengths, since they previously had the highest probability of reinfection (Fig. 4a and b). This implies that avoiding re-infections has a much more beneficial effect on the ec-network with respect to a random network. Even for the ec-network, without reinfections the incidence is reduced even at low coverage levels. For a treatment coverage of 10%, the annual number of treatments required is less than half of the treatments in the first scenario.

In the corresponding homogeneous-mixing model (SIR-type with population turnover: see equations 8–15) the annual number of treatments initially rises with increasing coverage before declining (Fig. 4c, both for simulations on networks and the homogeneous mixing solution, see equation 15). As in the first scenario, the homogeneous-mixing model predicts elimination of infection at a low coverage.

## Discussion

To our knowledge we present the first dynamic network model for the study of TasP of HCV. It has stationary features in line with real networks (exponential degree distribution, positive clustering and positive assortativity) while allowing for entry and exit of PWID. As recognised by others (e.g. refs 7 and 8) a dynamic network model is essential for studying transmission and prevalence of long-term infections like HCV, where duration of infection and duration of injecting behaviour are comparable. This cannot be captured in a static network model^12^. As Fig. 2a and b clearly show, network models and a homogeneous-mixing model differ greatly in the relationship between prevalence of infection and incidence of infection and reinfection. Consequently the models predict very different impacts of TasP. When the injecting-partnership network is represented realistically, TasP can greatly reduce the prevalence of infection in PWID, even in the absence of post-treatment behaviour change, but a given reduction requires greater coverage than predicted by a homogeneous-mixing model.

Importantly, the impact of TasP depends on the scale of the intervention – i.e. the coverage of diagnosis and treatment, in all studied scenarios and contact patterns. An intervention with low coverage has relatively little benefit since it does little to reduce the prevalence of infection and so there is rapid reinfection of patients who are successfully treated. However, if the intervention has sufficiently-large coverage then it produces a substantial reduction in the prevalence of infection, leading to reductions in the incidence of new infections and reinfections. This means that a higher coverage actually results in fewer treatments being required overall. Similar results have been found for other infectious diseases, including gonorrhoea^24^, MRSA^25^ and Ebola^26^.

Comparison of effectiveness of TasP on different networks shows that the shorter the average path-length, the higher the frequency of reinfection, and the greater the number of treatments required to reduce prevalence (Fig. 2a–c). This intuitive result can be explained more rigorously by a lower endemic threshold for skewed networks (with heterogeneous mean-field theory^27^), which holds also for slowly evolving networks^28^. The relationship holds despite an adjusted lower infection rate for exponential networks (see Table 1). With respect to our novel exponential-clustered network, the widely used Erdös-Renyi random network largely overestimates effectiveness of TasP because of its long average path-length, which makes it an unsuitable approximation. In contrast, an unclustered network under-estimates the effect of TasP, although the difference is less pronounced, in line with more theoretical studies^29^ and ref. 30.

The dynamics of reinfection after treatment are a major determinant of the impact of TasP for HCV in PWID. There are individual- and population-level aspects to these dynamics: the risk of reinfection depends upon both the behaviour of individuals who have been treated (i.e. if they inject drugs and the safety of their injecting practices if so) and the prevalence of infection in their injecting partners. This has consequences for the relationship between the scale of the TasP intervention and its effectiveness, and also for potential synergies arising from combining interventions.

At the individual level, if PWID who have been treated for HCV change their behaviour to reduce their risk of reinfection (as evidence suggests^13–17^) then this greatly increases the impact of TasP. We do not have estimates for our population so we compared two scenarios. In light of the variation in reported rates^18^ we recommend that post-treatment behaviour-change should be evaluated more thoroughly in relevant population groups, along with interventions to promote it.

At the population level, the greater the coverage of TasP the greater the reduction in prevalence and therefore the greater the reduction in reinfection risk – which in turn contributes to reducing prevalence. In addition, combining TasP with other interventions, like opiate substitution therapy (to assist people in stopping injecting, so reducing the number of susceptible and infected individuals in the network) and needle exchange programmes (to reduce the infection risk of injecting) could be synergistic^31^, greatly reducing the burden of HCV. The effectiveness of TasP may be increased by using “find and treat” services (e.g. ref. 32) for PWID to identify infections earlier to reduce the timespan in which they can transmit to others. (Furthermore, the acceptability of directly-acting antiviral treatment for HCV – which is far greater than previous treatments, which have unpleasant side-effects – could be effective in encouraging PWID to engage with health services.)

**Figure 5:**
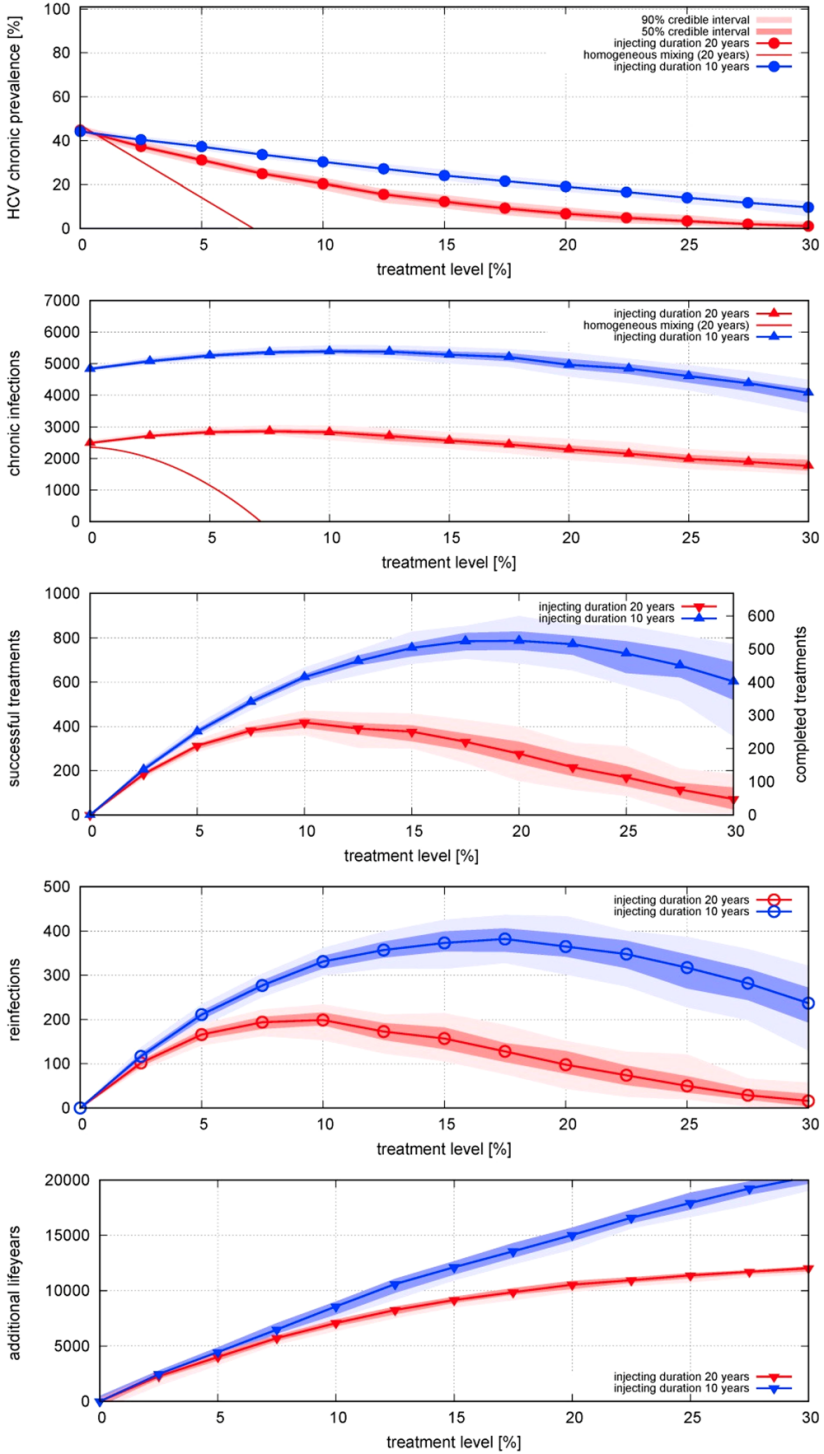
Comparison of injecting career duration of 20 years (red) and 10 years (blue) for the ec-network on the effect of different treatment rates on equilibrium (**A**) prevalence of HCV infection; incidence per year of (**B**) chronic infection (including arising from reinfection), (**C**) cured by successful treatment, (**D**) reinfection; and (**E**) life-years gained, for treatment rates varied from 0 to 30% per year. For the shorter injecting career duration, the number of individuals entering and leaving is twice as high (100/year) for a system of the same size (1000 individuals). The infection rate has been fitted to obtain 43% prevalence of chronic infection in the absence of treatment (*α* is 1.3 times the one for 20 years). For an infection duration of 10 years (close to the assumption in ref. 22), treatment is much less effective in reducing prevalence.

To inform policy decisions, it is necessary to understand the characteristics of the particular population of interest. We conducted a survey of PWID in London to design and parameterize our model. It is important to note that some key parameter values derived from the empirical study are different from those used in other modelling studies. With an average age of 44 years the cohort is older than the average age of 26 years used in ref. 6. As we showed in Fig. 5, injecting duration is a key parameter affecting the effectiveness of TasP, since it determines turnover and infection rate (assuming a given population size), on which TasP depends sensitively. As with all egocentric surveys, our empirical information on the structure of the network of PWID is limited, and we hope that our demonstration of the importance of having information on network clustering, degree distribution and population turnover will stimulate further empirical study to obtain this information, and on population turnover and changes in contacts over time. Although studies mapping networks of contacts are challenging, we showed that network structure is an important determinant of the effectiveness of TasP and decision-makers need to be aware of the uncertainty that arises from not having detailed information on network structure: all of the model networks that we examined are consistent with the data from our survey, highlighting the value of empirical study of the network. In addition, we recommend that those researchers who have conducted network studies and have detailed data available (e.g. refs 7, 8) analyse those data using dynamic rather than static networks.

In conclusion, treatment of PWID for HCV can produce substantial reductions in the prevalence and incidence of infection and consequent burden of disease, resulting in substantial life-years gained. This is the case even in the absence of post-treatment behaviour change reducing individual risk of reinfection and subsequent onward transmission. Nevertheless, it is important to realise that reinfection risk is a key determinant of the impact of TasP, and that reinfection risk depends upon both individual-level behaviour and the scale of the intervention applied to the population, and that small-scale trials are suitable only for measuring the individual-level component. Finally, it is important for policy-makers to know that commonly-used compartmental models (e.g. ref. 33) overestimate the benefits of TasP – in reality much larger interventions may be required to achieve the anticipated health benefits – and to improve assessment of the potential impact of TasP in different populations of PWID we recommend further empirical study of injecting-partnership networks, and use of dynamic network models to represent those networks realistically.

## Methods

### Survey of people who inject drugs

We conducted a survey of PWID asking about demographic information and injecting behaviour, including numbers of injecting partners in last 6 months, the last year, the last five years and lifetime, and the length of time spent injecting drugs. The survey was conducted alongside community HCV testing offered at drug treatment services, homeless hostels, homeless day centres and congregate settings in London by the Hepatitis C Trust mobile screening van between June 2015 and January 2016. Written informed consent was obtained from all study participants. The study conforms to the ethical guidelines of the 1975 Declaration of Helsinki; all protocols were approved by the Brent NRES Committee, London (Ref: 13/LO/0077); methods were carried out in accordance with the relevant guidelines and regulations; informed consent was obtained from all subjects.

The survey of PWID in London had a participation rate of 76% (97/127). Average age was 43.8 years, (range 25–67, interquartile range 38–50), and the average duration of injecting career was 17.5 years (range 0–44, interquartile range 10–23). 56% of respondents reported ever having shared syringes, and 80% reported ever having shared paraphernalia. Injecting frequency was on average 18 times per week (range 1–70). (Although our data come from a study of the relevant population, often modelling studies of HCV in PWID have assumed a shorter duration of injecting career, so we examined the effect of assuming a shorter average injecting career (Fig. 5): unsurprisingly, a higher rate of turnover results in a higher incidence for the same prevalence, and means that a given treatment level results in a smaller reduction in prevalence, and with more treatments required.)

### Mathematical modelling of injecting contact networks

Based on information from the survey and evidence from the literature, we developed an algorithm by which simple behavioural rules of PWID produce a dynamic injecting-partnership network (termed “exponential-clustered network”) with characteristics found in real networks: i.e. an exponential degree distribution^20, 22^, clustering^9, 23^, as well as positive assortativity (degree correlations)^20^, and a short average path-length^34^ between two people (see Table 1 and Fig. 1). These features affect epidemic spreading, which has been studied on different types of networks in the complex networks literature, often with the so-called heterogeneous mean-field approximation^27^, quenched mean-field approximation^29, 30^, or spectral methods^35, 36^. To understand the effects of both degree distribution and clustering, we used networks with simpler characteristics for comparison: a skewed unclustered network (with the same exponential degree distribution) and a random network. All networks have a stationary degree distribution, stationary number of people and stationary number of partnerships. The networks are dynamic since people (nodes) enter and exit, entering ones form new sharing partnerships (links) and exiting ones break theirs.

### Network-generating algorithm: exponential-clustered (ec) network

The algorithm by which PWID find injecting partners operates as follows:

(i) A person entering the network selects a partner: for each potential partner, the probability of being selected is proportional to the potential partner’s current number of partnerships.
(ii) People can also leave the network after a given duration, such that the system has a constant number of people (nodes) and partnerships (links). If a person leaves then all their partnerships break.
(iii) The total number of partnerships in the network is constant. New partnerships are therefore created as follows: a link (i.e. partnership) is selected at random, and one of its partners is chosen to receive an additional link (this means that every person is picked at random, with probability depending on its number of partnerships).
(iv) This person’s new partner is picked at random among the first partners’ partners. Only in the rare cases where this is not possible (e.g. in a disconnected couple), a partner in the whole network is chosen as in (ii). This selection method leads to both positive degree correlations (assortativity) and clustering (see Fig. 1). In the case of zero or one partner, this is not possible, and a partner is again picked with probability proportionate to its number of partnerships.
(v) People exit the system after a given time. If an exiting person leaves a partner unconnected then this former partner will form a new partnership, and a partnership picked at random is broken, to keep the number of partnerships constant.

The algorithm generate an exponential degree distribution numerically and theoretically^19^, in line with evidence^22^ and irrespective of the initial topology (see inlay right in Fig. 1c). Step (iv) generates in addition a positive clustering coefficient (measured in the transitivity function of the R package *igraph*; assortativity coefficient is calculated as defined in ref. 37). This strengthens the plausibility of the simple network formation hypotheses used here, that people tend to find partners locally, as well as connect preferably to the well-connected.

### Network-generating algorithm: exponential unclustered network

A skewed unclustered network with the same exponential degree distribution was generated by the following alternative step (iv):

(iv-a) A partner’s partners aren’t given priority and the partner is picked with probability depending on the person’s current number of partnerships.

### Network-generating algorithm: random network

A random network was obtained by changing step (i) so that a new sharing partner is chosen at random. This means that the Erdös-Renyi network is recovered, which has binomial degree distribution (stationary under entry and exit), and a longer average path-length than both the exponential-clustered and the exponential network.

### Modelling of transmission and treatment

To compare transmission dynamics on the three networks, we consider networks with the same number of nodes, entry and exit rate and same average degree (Table 2). Each of them has its transmission rate calibrated to produce HCV prevalence in PWID in London of 43%;[Aldridge *et al*. submitted] seroprevalence in PWID in London was 53%, which is similar to national estimate of 50%^38^.

**Table 2:** Demographic and natural history parameter values.

Parameter	Value	Reference
Infection rate β (per partnership per month)	ec network: 0.053; exponential network: 0.053; random network: 0.068	Fitted to obtain 43% prevalence
Treatment rate α (varied in scenario analysis)	0–30%	(scenarios)
Average number of partners at one point in time	3.7	Survey reported in this study
HCV prevalence without treatment	43%	[Aldridge *et al*. submitted]
Duration of injecting career	20 years	This study
Rate of new entrants per year	5%	This study
Disease progression rates	(varies by age)	40
Death rates	(life tables from Office for National Statistics, age-dependent)	39
Incremental annual death rate for PWID	0.01	6
Time period considered	20 years	

We study two scenarios: one where cured individuals can get reinfected, and one where individuals change their behaviour to avoid reinfection; the former is a susceptible-infected-susceptible (SIS)-type transmission-dynamic model and the latter a susceptible-infected-recovered (SIR)-type model, both with entry and exit. These scenarios were simulated on each of the networks. In discrete time, the succession of events is

1. Entry of new PWID
2. Formation of new partnerships
3. Infection of susceptible individuals (with rate *β* per contact with each infected partner)
4. Exit of people from the network
5. Treatment (of infecteds with probability *α*)

The system converges to an equilibrium prevalence level, although, as with any finite system, the dynamics will eventually stochastically reach a state of zero prevalence, from which it cannot rebound^21^. For our model, this probability is negligible in the considered timespan, unless equilibrium prevalence is close to zero.

We also compare the effect of the different scenarios in terms of life-years gained (both via increased life expectancy per treated case and avoided infections), using age at infection, life expectancy for UK^39^, and HCV progression parameters from^40^. This gives estimates on the number of cases of liver disease of each treatment scenario. The method is detailed at the end of the Methods section.

### Comparison with homogeneous-mixing model with reinfection (SIS-type)

If instead of a network a system of infinite size and homogeneous mixing is assumed, the model follows the equations,

1 $$\frac{dS}{dt}=-\,\beta \frac{SI}{N}+\alpha I+\mu {N}_{init}-\frac{{\rm{1}}}{\tau }S$$

2 $$\frac{dI}{dt}=\beta \frac{SI}{N}-\alpha I-\frac{{\rm{1}}}{\tau }I$$

where *β* is infection rate, *α* treatment rate, *μ* entry rate of susceptibles, *τ* time spent in the network, *S* susceptibles, *I* infecteds and *N* system size. It has as stationary state the equilibrium system size *N* = *μτN*_*init*_ and *μ* = 1/*τ* and for the fraction *s* of susceptibles and *i* infecteds

3 $$s=\frac{\alpha +{\rm{1}}/\tau }{\beta }$$

and

4 $$i={\rm{1}}-\frac{\alpha +{\rm{1}}/\tau }{\beta }={\rm{1}}-\frac{{\rm{1}}}{{R}_{{\rm{0}}}}$$

where

5 $$s+i={\rm{1}}$$

For no endemic state with *i* > 0 can persist, and *R*_0_ is the epidemic threshold. The rate of new infections and treatments is

6 $${\rm{infections}}=\beta si=(\alpha +\mu )({\rm{1}}-\frac{\alpha +\mu }{\beta })$$

7 $${\rm{treatments}}=\alpha i=\alpha ({\rm{1}}-\frac{\alpha +\mu }{\beta })$$

This however neglects the time to infection and therefore yields a much higher prevalence for a given infection rate. The epidemic threshold *R*_0_, above which endemic states can sustain would be finite even in that case, and treatment as prevention would appear much more effective than on any of the three studied networks.

The comparison to the results of differential equations is has some limitations since the studied networks are evolving, and time spent in the network plays an important role, which increases with average pathlength.

### Comparison with homogeneous-mixing model without reinfection (SIR-type)

The rates of entry and exit equal i.e. $\mu =\frac{{\rm{1}}}{\tau }$. The compartments are now susceptibles *s*, infecteds *i* and recovered *r* (which are not at risk of infection). They follow the equations,

8 $$\frac{ds}{dt}=-\,\beta si+\mu -\frac{{\rm{1}}}{\tau }s$$

9 $$\frac{di}{dt}=\beta si-\alpha i-\frac{{\rm{1}}}{\tau }i$$

10 $$\frac{dr}{dt}=\alpha i-\frac{{\rm{1}}}{\tau }r$$

with

11 $$s+i+r={\rm{1}}$$

equilibrium prevalence level as a function of treatment rate is then

12 $$i=\frac{\mu (\beta -\alpha -\mu )}{\beta (\alpha +\mu )}$$

13 $$s=\frac{\alpha +\mu }{\beta }$$

and the rate of new infections and treatments is

14 $${\rm{i}}{\rm{n}}{\rm{f}}{\rm{e}}{\rm{c}}{\rm{t}}{\rm{i}}{\rm{o}}{\rm{n}}{\rm{s}}=\beta si=\frac{\mu (\beta -\alpha -\mu )}{\beta }$$

15 $${\rm{t}}{\rm{r}}{\rm{e}}{\rm{a}}{\rm{t}}{\rm{m}}{\rm{e}}{\rm{n}}{\rm{t}}{\rm{s}}=\alpha i=\alpha \frac{\mu (\beta -\alpha -\mu )}{\beta (\alpha +\mu )}$$

### Calculation of averted disease progression and averted infections

**Figure 6:**
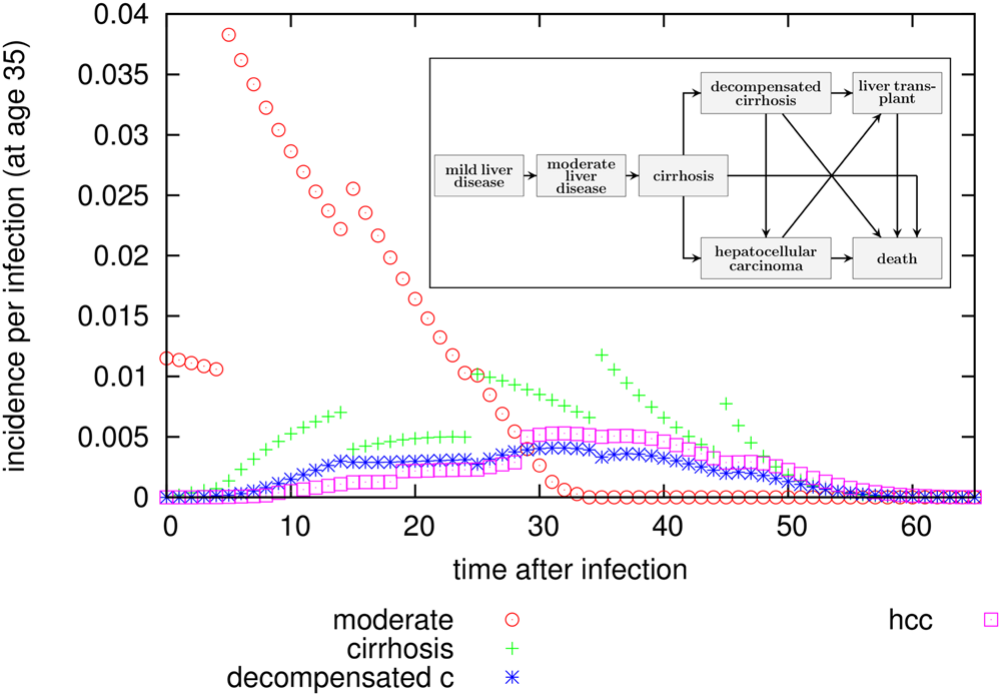
Probability for incidence of progression states as a function of time after infection. These probabilities are calculated separately for each infection age (in this figure this is 35 years of age), since we use age-dependent disease progression values^40^ as well as mortality data^39^. From these values, cumulative incidences as a time after infection can be calculated, and from that the average number of life years lost (again as a function of time of infection), which allows to calculate an estimate on the number of life-years saved at system-level via comparison of two scenarios. The lines of points are discontinuous because disease progression parameters are age-dependent and sometimes differ strongly between age-groups.

Separately from the transmission dynamics, we modelled progression of a typical HCV infection to moderate liver disease, cirrhosis, decompensated cirrhosis and hepatocellular cancer, as shown in the inlay in Fig. 6. We used values based on a retrospective study^17^, which calculated age-dependent disease progression rates, which are comparable but more detailed and recent than the ones^33^ used in the NICE guidelines. In addition, we used life expectancy data from the UK^39^ to calculate the progression probability of a typical infection, separately for each infection age (see as an example Fig. 6 for age 35). We assumed the infected cohort was 70% male, and that there was an incremental death probability of 0.01 per year for PWID compared to the general population, as in^33^.

For a given infection age a0, the probability to be in progression state “moderate liver disease” can be calculated with the so-called master equation:

$${\rho }_{moderate,a0,age}={\rho }_{mild,a0,age-1}{\gamma }_{moderate,age-1}-{\rho }_{moderate,a0,age}{\gamma }_{cirrhosis,age}-{\rho }_{moderate,a0,age}{\gamma }_{death,age}$$

and for later progression states accordingly. From this probability, annual incidences (and cumulative incidences) can be calculated, following

$${i}_{{moderate},age}={\rho }_{mild,age-1}{\gamma }_{{moderate},age-{1}}$$

**Figure 7:**
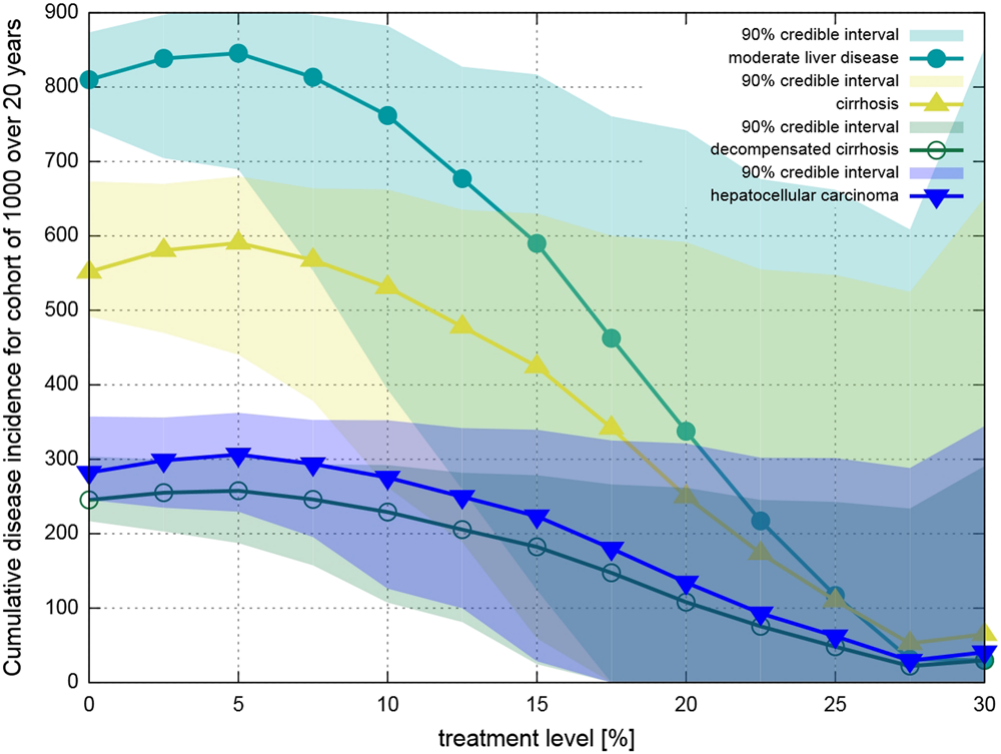
Cumulative number of cases of liver disease progression states as a function of treatment level, calculated for a population of 1000 PWID for infections occurring over a 20 year timespan. The credible intervals given here are for 100 simulations; disease progression follows the age-dependent probabilities of^40^ exactly. The lines are discontinuous because disease progression parameters are age-dependent and sometimes differ strongly between age groups.

From the transmission-dynamic model, we then obtained time series of infections and successful treatments, as well as age at infection and treatment. Using this and the typical evolution of one infection, we calculated the number of cases occurring from a given treatment scenario (see Fig. 7). Comparison of scenarios allows calculation of the number of life-years saved, as well as averted cases of moderate liver disease, cirrhosis, decompensated cirrhosis ad cancer (see Figs 2e and 4e). The disease cases may arise long after the individual has left the network; the calculation considers the time period until the end of life of every person who has ever been in the network.
